# Comparing the Influence of Residual Stress on Composite Materials Made of Polyhydroxybutyrate (PHB) and Amorphous Hydrogenated Carbon (a-C:H) Layers: Differences Caused by Single Side and Full Substrate Film Attachment during Plasma Coating

**DOI:** 10.3390/polym13020184

**Published:** 2021-01-06

**Authors:** Torben Schlebrowski, Rachida Ouali, Barbara Hahn, Stefan Wehner, Christian B. Fischer

**Affiliations:** 1 Department of Physics, University Koblenz-Landau, 56070 Koblenz, Germany; wehner@uni-koblenz.de; 2 Department of Material Analysis, University of Applied Sciences Koblenz, RheinAhrCampus, 53424 Remagen, Germany; r_ouali@hotmail.de (R.O.); hahn@hs-koblenz.de (B.H.); 3 Materials Science, Energy and Nano-Engineering Department, Mohammed VI Polytechnic University, 43150 Ben Guerir, Morocco

**Keywords:** biopolymer polyhydroxybutyrate (PHB), a-C:H layers, gradual film deposition, stress release phenomena, chemical surface environment

## Abstract

Polyhydroxybutyrate (PHB) is a bio-based, biodegradable and commercially used polymer, which in its native form is unfortunately not generally applicable. A widely used technique to adapt polymers to a wider range of applications is the surface modification with amorphous hydrogenated carbon (a-C:H) layers, realized by plasma-enhanced chemical vapor deposition (PE-CVD). However, this process creates intrinsic stress in the layer–polymer system which can even lead to full layer failure. The aim of this study was to investigate how the carbon layer is affected when the basic polymer film to be coated can follow the stress and bend (single side attachment) and when it cannot do so because it is firmly clamped (full attachment). For both attachment methods, the a-C:H layers were simultaneously deposited on PHB samples. Ex-situ characterization was performed using a scanning electron microscope (SEM) for surface morphology and contact angle (CA) measurements for wettability. In addition, the stress prevailing in the layer was calculated using the Stoney equation. Diffuse reflectance infrared Fourier transform spectroscopy (DRIFT) measurements were used to investigate the chemical composition of the coating surface.

## 1. Introduction

Today, polymers have become an integral part of everyday life and are indispensable in many fields of application. More than 360 million tons have been produced in 2019 worldwide [1]. Since many polymers are based on crude oil, the growing demand for plastics not only leads to a higher oil consumption, putting further strain on fading reserves, but also further pollutes the environment through disposal (in 2017 more than 302 million tons of plastic were wasted) as degradation of these materials and their composites is usually difficult and protracted [2,3]. An alternative candidate to replace conventional polymers is the biodegradable polymer polyhydroxybutyrate (PHB), which is produced by fermentation [4]. Nevertheless, it has comparable material characteristics to conventional polymers; e.g., formability, elasticity, chemical resistance, but also low hardness and poor mechanical properties [5,6,7]. Parallel to the known limitations of polymer surfaces, the aspect of biodegradability also comes into play here. This occurs mainly via the surface and thus leads primarily to a lower durability.

Thin amorphous hydrogenated carbon (a-C:H) films are often deposited on polymers to customize their surface properties [5,8,9,10,11,12,13,14]. The application of the carbon layer can be realized by chemical vapor deposition (CVD), frequently amplified by radio frequency plasmas (RF-PECVD) [9,15]. An advantage of this method is that the substrate temperature is kept low and substrates such as polymers that are not conductive can be coated [10,11,13,16]. The resulting a-C:H network is composed of both sp^2^ (π- and σ-) and sp^3^ carbon configurations. Here, the sp^3^ matrix of carbon and hydrogen contains small sp^2^ clusters limited to short chains [8,9,17,18]. Not only the plasma parameters during the coating process, but also the thickness of the coating itself determine the bonding ratio of the carbon atoms (sp^2^-to-sp^3^ ratio) and thus, together with the hydrogen content, the chemical composition of the coating [9,11,13,16,18]. This allows the physical properties to be controlled and adjusted to desired specifications (e.g., inertness, hardness, friction, durability) [19]. However, it is problematic that these films have an internal stress which increases with increasing film thickness and can assume values of several GPa at high thicknesses [20,21,22]. This can ultimately result in layer failure [11,13,16]. In addition, there are various ways in which workpieces are attached for coating in the industry. For example, catheters are attached to a single fixed point [23,24]; free-hanging or complete attachment is conceivable in the present films. Therefore, the influence of fixation and stress on the a-C:H coatings produced here is investigated.

In this study, a-C:H layers are deposited on “fixed” and “free” PHB film samples and differences between the resulting carbon layers are investigated. The RF-PECVD process was used to produce the coatings; acetylene (C_2_H_2_) served as carbon source. Surface morphology was examined ex situ by scanning electron microscopy (SEM) and diffuse reflectance infrared Fourier transformation (DRIFT) measurements were performed for the chemical composition of the carbon coatings. Using the modified Stoney equation, the stress prevailing in the layers was determined. Furthermore, the surface wettability was analyzed with contact angle (CA) determinations.

## 2. Materials and Methods

### 2.1. Sample Preparation and Film Deposition

**Figure 1:**
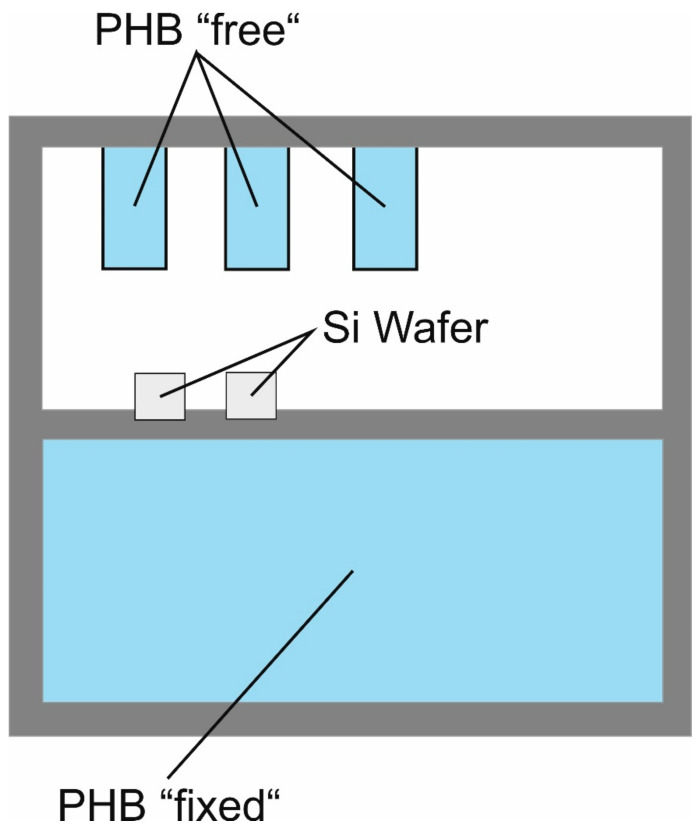
Samples holder set-up. The “free” polyhydroxybutyrate (PHB) samples sized 1 × 2 cm at the top are free on three sides; the size of the “fixed” sample is 5 × 10 cm, which is clamped on all four sides.

Industrial grade polyhydroxybutyrate (PHB) foils were purchased from Goodfellow (Bad Nauheim, Germany) as substrate. The 50 µm thick foils (30 × 30 cm) were cut for the experiment to 5 × 10 resp. 1 × 2 cm samples and fixed on in-house designed aluminum holders. The samples were placed together but in two different ways on the holder: (a) the strips measuring 1 × 2 cm were placed in the holder in such a way that they were only fixed on one side and free on three sides. This should enable the samples to freely follow the stress prevailing in the layers. They are described as “free” in the following. (b) The second variant was pieces of foil that were firmly clamped on all four sides, which means that the layer must adapt to the substrate. These are referred to as “fixed” in the following. A scheme of an equipped sample holder is shown in Figure 1. This set-up ensures that the same plasma parameters and conditions are available for both deposited layers.

A high-frequency (RF, 13.65 MHz) plasma source (Copra DN 400, CCR GmbH, Troisdorf, Germany) in a vacuum process chamber was used for the deposition of the coatings [14]. The plasma process was divided into two steps and is described in detail elsewhere [13]. Therefore, only main steps are given here: application of an oxygen plasma (O_2_, 10 min) as a pretreatment for the cleaning and activation of the polymer surfaces and an C_2_H_2_ plasma for the coating with r-type a-C:H layers itself [14,16,25,26]. The distance between plasma source and samples was continuously 275 mm. With variation of C_2_H_2_ plasma coating time (rate 10 nm cm^−1^), four different a-C:H layer thicknesses up to 170 nm were realized (10, 50, 120 and 170 nm) and an only O_2_ treated holder was used as “new” reference. In addition, silicon wafers (Silicon Materials, Kaufering, Germany) which were covered half with aluminum foil, were mounted on each sample holder (Figure 1). Profilometry was used to measure the layer thickness and curvature of the applied a-C:H coatings (Dektak 3, Veeco Instruments Inc., Plainview, NY, USA).

### 2.2. Stoney Equation

During the deposition of layers on a sample, the workpiece is subjected to a certain strain referred to as stress. Two types of stress are distinguished: (a) tensile stress. Here a coated sample will bend inwards under the tension that the layer exerts on the material. (b) The so-called compressive stress. In order to maximize the surface of the applied layer, the sample bulges outwards and thereby the layer is stretched and minimizes its contact with the substrate surface [19]. The Davis model can be used to explain the compressive stress in a-C:H films, where their structure is explained by two competing processes. The subplantation of high-energy ions enhances the compressive stress by compression of the a-C:H film. As a counter process, ions whose energy exceeds the energy required for subplantation reduce the compressive stress. This happens because they release their energy as a thermal peak in the layer, which reduces the compressive stress [27]. This stress limits the possible layer thickness to values from several hundred to several thousand nm before the applied layers break due to the stress and detach from the substrate surface [9]. For a stable, adhered layer the force exerted by the stress must be absorbed by adhesive forces. The stress in a substrate-layer system can be calculated by a modified form of the Stoney equation. By measuring the curvature radii of the sample prior to and upon coating, this equation is able to calculate the stress produced in the layered composite [28].

(1) $$\sigma_{f}=\frac{Y}{1-\nu_{s}}\frac{h_{s}^{2}}{6h_{l}}\Delta K$$

The unit of stress is Pascal. Here Δ*K* is the difference between the inverse of the radii of curvature of the coated and uncoated samples [28]. The curvature radii were measured by profilometry; the value for the biaxial modulus of elasticity, *Y*, and the Poisson’s ratio, *ν*_s,_ are taken from the manufacturers’ data sheets. The parameter *h*_s_ is the substrate thickness, *h*_l_ the applied layer thickness. However, the Stoney equation can only be applied if some conditions are observed: firstly, the sample must have a rectangular shape (a < b; length dimension here a = 1 cm, b = 2 cm). The small side must be much larger than the substrate thickness (*h*_s_ << a; here *h*_s_ = 50 µm) [28]. Secondly, the thickness of the applied layer must be much smaller than the thickness of the substrate: *h*_s_ >> *h*_l_ [28].

If the resulting value is negative, compressive stress is present; i.e., the substrate is curved outwards. If the obtained value is positive, the substrate will bulge inwards and tensile stress will be present. The present stress values have been calculated for three samples and each film thickness.

### 2.3. Layer Analysis

Three techniques were used to analyze the samples obtained. In order to investigate the surface morphology, scanning electron microscopy (SEM) measurements were performed on a SEM515 from Philips (FEI Company, Amsterdam, The Netherlands). Since PHB is a poor conductor and the deposited a-C:H layers are also not necessarily conductive, a 7–10 nm thick gold layer was sputtered onto the sample surface to prevent charging effects. At least three different locations on each sample surface were examined (7 kV, 20 mm) to check accuracy. Contact angle (CA) measurements were performed to check the surface wettability (OCA 15 plus, DataPhysics Instruments GmbH, Filderstadt, Germany). A 1 μL drop of water (HPLC grade, CHEMSOLUTE^®^, Th. Geyer GmbH & Co. KG, Renningen, Germany) was automatically dispensed on the surface and CA values were measured for at least five different locations (ambient air, room temperature (RT)). The left and right sides of the drop were measured and averaged for each location. The chemical composition of the a-C:H coatings was analyzed by diffuse reflectance infrared Fourier transform (DRIFT) spectroscopy (Shimadzu IRPrestige-21 incl. diffuse reflectance measuring unit DRS-8000, Kyoto, Japan) at RT and ambient conditions [29,30]. Two different spectra were taken (each at min. three different locations): first, an overview spectrum covering 450–4000 cm^−1^ (resolution 4 wavenumbers, 100 repetitions) to investigate which regions were affected as a result of the different layer thicknesses. Subsequently, a more detailed measurement (resolution 1 wavenumber, 300 repetitions) was performed in the C-H stretching region 2800–3050 cm^−1^ [31,32]. For the DRIFT studies, the respective PHB sample treated with O_2_ plasma was used as the reference for the deposited a-C:H layers, because each sample was pretreated with an O_2_ plasma. The analysis of the spectra obtained was performed with the IR Solution—FTIR Control software (software version 1.30, Shimadzu Corporation, Kyoto, Japan): a multi-point baseline insertion and fine-tuning (smoothing) with software-integrated manipulation tools. These two steps only change the appearance of the graph, but not the contained information (for details see IR evaluation curve fitting available in the Supplementary Materials).

## 3. Results and Discussion

### 3.1. Stress

**Figure 2:**
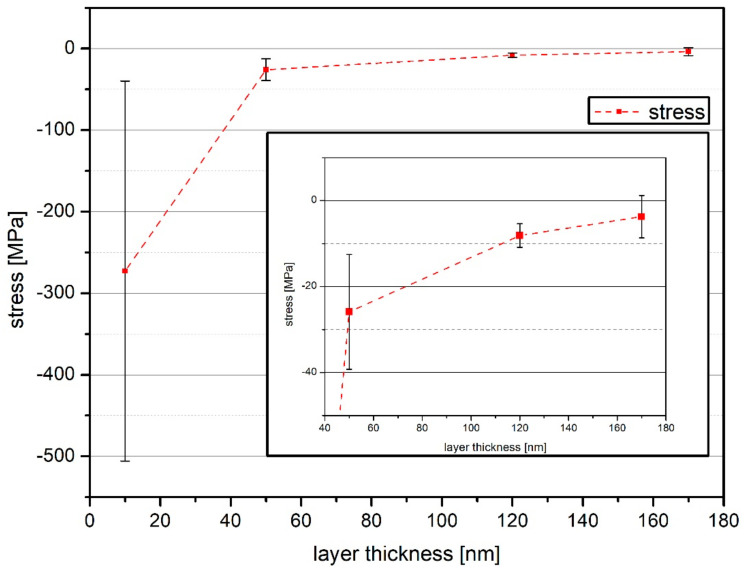
Calculated compressive stress for a-C:H layers with thicknesses from 10–170 nm on PHB (the dashed line only indicates a trend). The region for the later depositions is enlarged (inset) for better visibility.

Figure 2 shows the stress values of the analyzed samples calculated with the Stoney’s equation. For the sample with 10 nm a-C:H layer thickness, a high compressive stress of 270 MPa was calculated. The error bar is nearly in the same region. This high value for stress and error may be due to the fact that layer growth was just starting. The formation of an interlayer (a mixed phase of eroded substrate material and the a-C:H layer deposited by the plasma [14]) was just beginning and no stable or closed layer had formed yet. In addition, the Stoney equation divides by the only small layer thickness and the raw foils already showed a slight curvature at the beginning, which can cause the high values for the calculated stress and error. Due to the high stress value of the 10 nm, further results were enlarged (inset in Figure 2). When reaching a layer thickness of 50 nm the stress was strongly reduced compared to the thin 10 nm layer. The compressive stress here was only 25 MPa. As the coating thickness increased, the stress was reduced further and further until it reached a value of only 4 MPa at the final coating thickness of 170 nm. A possible explanation for the decrease in stress is the bonds present in the layer. From previous investigations with the same conditions it is known that the layer deposited on PHB is strongly dominated by sp^2^ in the thickness range of 100–200 nm [16]. The sp^2^ dominated layers are known to be less stressed than sp^3^ dominated ones [33,34] and therefore, the result of the determined stress would be in line with the previous measurements [16]. Another possible explanation is stress release phenomena like cord-buckling of layer failure [35,36].

### 3.2. Surface Morphology and Wettability

**Figure 3:**
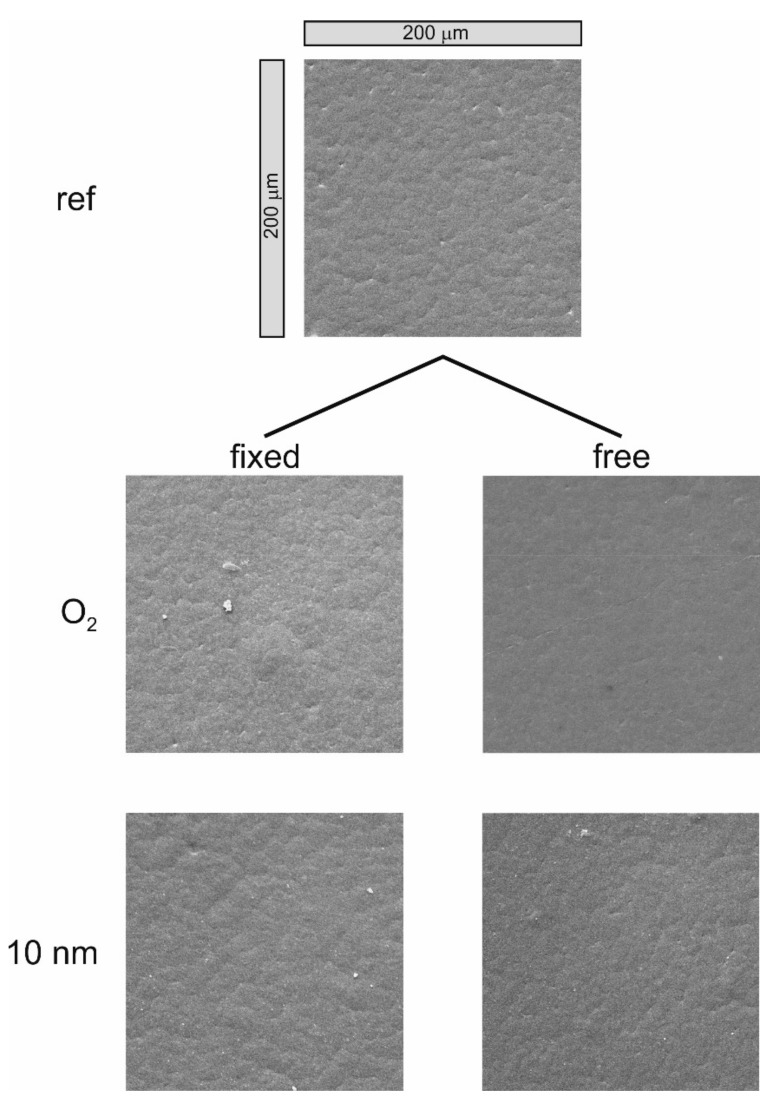
SEM images of the raw PHB, O_2_ treated samples and 10 nm layer thickness for the “fixed” and “free” samples.

**Figure 4:**
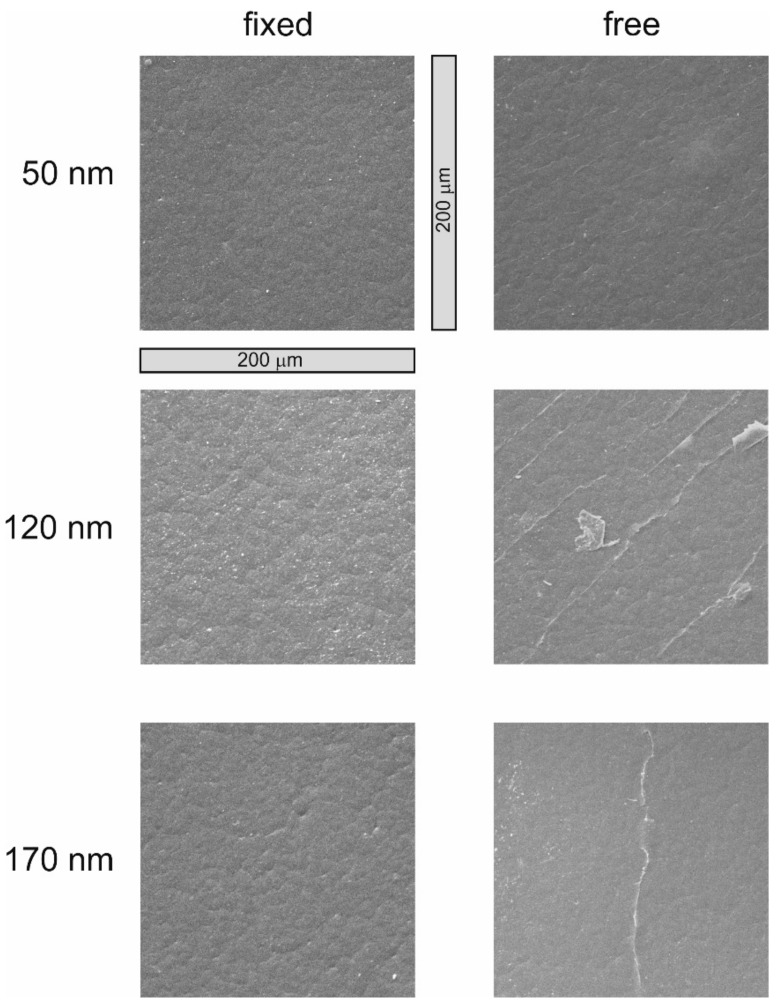
SEM analysis of a-C:H layers on PHB with thicknesses from 50, 120 and170 nm. On the left are the “fixed” samples and on the right the “free” ones.

Figure 3 and Figure 4 show the SEM images of the untreated PHB, after oxygen treatment and after coating the polymer samples with increasing a-C:H layer thickness. On the left side are the “fixed” samples; on the right side the “free” samples. The thickness increases from top to bottom. The selected magnification for all images is 442× (full SEM images are available in the Supplementary Materials).

Figure 3 shows the untreated polymer, the O_2_ treated samples and the layer thickness of 10 nm. For the untreated polymer, the granular structure resulting from production is clearly visible. If the samples were treated with O_2_ plasma, the granular structure was still clearly visible for both samples. For the “fixed” sample it was even more pronounced than before, caused by the sputtering effect of the O_2_ plasma. If 10 nm a-C:H was deposited on the material, no differences were visible for the “fixed” and the “free” sample. In both cases, a layer formed on the structure of the PHB and gradually began to cover it.

Figure 4 shows the layer thicknesses of 50, 120 and 170 nm for both attachment types. In the 50 nm thick a-C:H layer, the first differences between the attachment methods can be seen. The “fixed” sample had a homogeneous, closed and adherent layer on the polymer surface. The 50 nm “free” probe, on the other hand, shows the so-called “telephone cord-buckling” effect in an initial stage [35,36]. The layer is still closed, but crimping and rippling are already visible. This is a stress release phenomenon and already known for PHB, but for thicker layers [16]. If the film was able to follow the layer as was the case in the free samples, the stress contained in the layer was apparently higher than if the layer had to adapt to the film. If the 120 nm thickness is considered, the a-C:H layer was still closed and homogeneous in the “fixed” samples, the telephone cord-buckling effect did not occur. The “free” sample, however, shows clear cracks in the layer. The stress had apparently increased to such an extent that the structure of the layer was no longer able to counteract and the layer was partially destroyed. If a thickness of 170 nm was reached, the layer was still stable and closed in the “fixed” samples. Cracks were still visible on the “free” specimen, and the layer thickness was not stable if the polymer sample had to follow the layer.

In general, the surfaces of “fixed” samples seemed a little rougher than those of “free” samples. In addition, it is noticeable that the “free” samples generally had a higher proportion of internal stresses and were more unstable than when the film was fixed and the layer had to adapt. The results of the Stoney equation show that the stress in the film decreased with increasing film thickness. This can also be seen in the SEM images of the “free” samples. The stress release phenomenon telephone cord buckling was an attempt by the system to reduce stress. This culminated in the breaking of the layer on the PHB foils, which released internal stresses. As a result, the stress calculated in the layer decreased.

**Figure 5:**
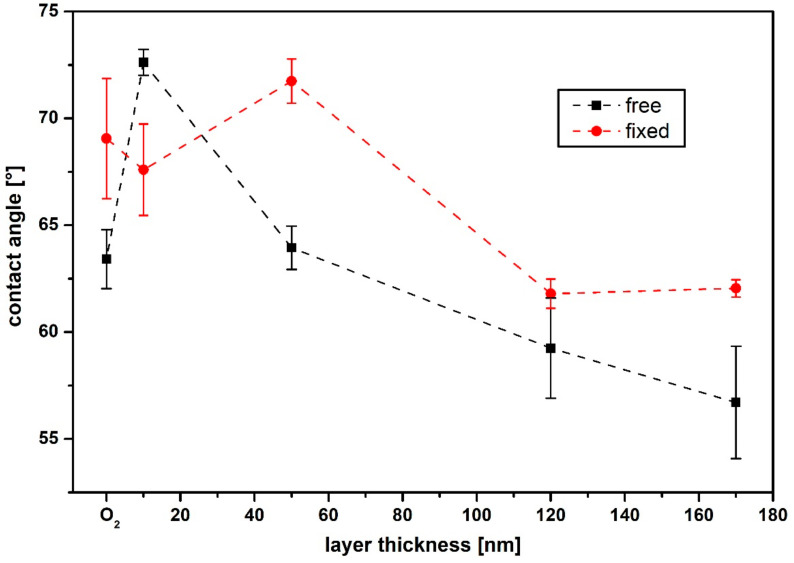
Contact angles of a-C:H coatings from 10 to 170 nm thickness on PHB. The red dots are the “fixed” samples, the black squares the “free” ones (the dashed line only indicates a trend).

Figure 5 shows the contact angles (CA) for the “fixed” and “free” samples for the different layer thicknesses. The wettability of the surface and the resulting CAs depended on three factors: first, the morphology of the surface [37,38,39] had an influence, then the hybridization states of the surface carbon atoms [17,40,41] and finally the chemical bonds present on the surface [42,43,44,45]. Thus, CA analysis can be used to investigate the chemical composition of the sample surface as well as its morphology, as these have an influence on the surface wettability [46].

Different carbon hybridization states led to a change of the contact angle at the sample surface [26,30]. Thus, for an sp^3^-dominated surface, the surface energy was higher compared to an sp^2^-dominated surface with lower polarity as a result of its stronger covalent nature [17,19]. Therefore, surfaces rich in sp^3^ have a reduced CA, since a polarity increase gives rise to smaller CAs and higher hydrophilicity [47]. If hydrogen saturates the free bonds on the surface, further interaction between the sample surface and fluid (which here was water) is suppressed by the strong C-H bonds [42,43,44]. In contrast, a less hydrogenated surface is more hydrophilic. If oxidation of the surface occurs, the formation of oxygen functionalities consequently leads to an increased attractiveness for water [45]. However, due to the demanding morphology of the “free” samples examined here, the CA values are only indicative and reflect a trend.

It can be seen that the angle for the O_2_ plasma treated “free” sample (black squares) with 63° differed from that of the “fixed” sample (red dots) with 69°. Compared to the untreated PHB sample, which had a CA of 75°, it can be seen that the oxidation of the surface led to a better water wetting and therefore the angle decreased. Applying a thin a-C:H layer of 10 nm on the “fixed” PHB sample led to a slightly reduced CA (68°). If the layer thickness increased to 50 nm, the value went up to 72°. This increasing CA cannot be fully explained at the moment and is part of further investigations. If the thickness of the layer increased, CA decreased. The CA for the “fixed” specimen was around 62° for both 120 nm and 170 nm film thickness.

If a very thin a-C:H layer is applied, the CA on the surface of the “free” sample increased noticeably to almost 73°, which was the maximum for the present layers. When the layer thickness was increased to 50 nm, this angle decreased again to 63°. This trend continued with increasing film thickness and reached its lowest value of 57° at 170 nm. Comparing the results of the “fixed” and the “free” samples, the CAs for the “fixed” samples from 50 nm on were significantly higher than for the “free” samples. Therefore, also with this measurement technique the layer changes if the material is either firmly clamped or can follow the layer during its coating.

### 3.3. Chemical Composition

DRIFT studies were performed to investigate whether the different methods of PHB film attachment caused a variation in chemical composition of the a-C:H layers. Since all samples were O_2_ plasma treated before, these samples were used as reference. Afterwards, the ascending layer thicknesses were recorded in order to determine the pure changes of the surface bonds through the a-C:H coating. The evaluation of the spectra was done on the basics of infrared spectroscopy [31] and based on previous results and the work of other groups [11,12,13,16,48,49,50,51,52,53].

**Figure 6:**
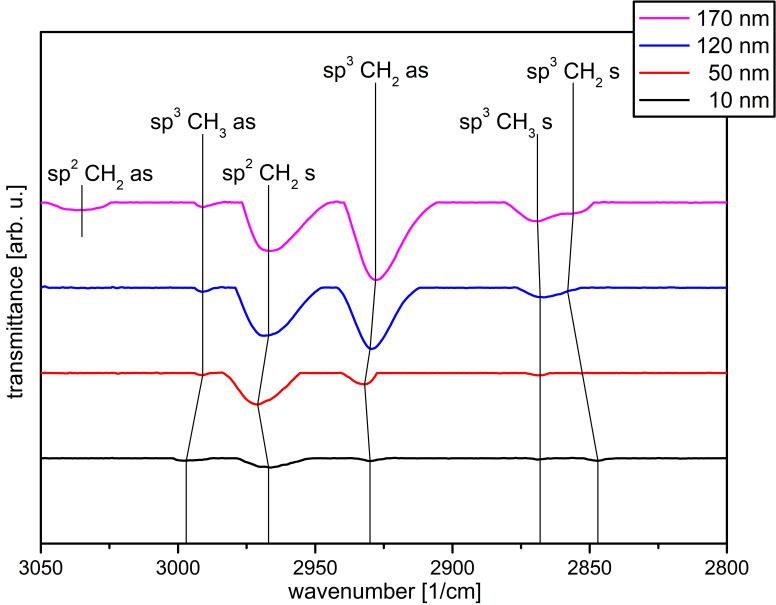
Evaluated DRIFT spectra of the “fixed” PHB samples coated with a-C:H from 10–170 nm in ascending order. The wavenumber is plotted against the transmittance in arbitrary units.

In Figure 6 the analyzed spectra for the a-C:H layers on the “fixed” PHB samples are plotted. For the 10 nm a-C:H layer five bonds are visible, which are only weakly formed due to the small layer thickness. The most prominent peak is the sp^2^CH_2_ symmetrical (s) peak at 2967 cm^–1^ [50]. Furthermore, two peaks at 2997 cm^–1^ asymmetrical (as) and 2868 cm^–1^ (s), belonging to the sp^3^CH_3_ oscillation as well as the peaks at 2930 cm^–1^ (as) and 2847 cm^–1^ (s) belonging to the sp^3^CH_2_ oscillation, are visible [50,51]. With 50 nm a-C:H the peak associated with the sp^3^CH_2_ (s) oscillation disappears. The sp^3^CH_3_ (as) peak shifts to smaller wavenumbers at 2991 cm^–1^. With this backward shift, a smaller C to H binding energy is indicated and therefore the C to H bond distance for the associated CH_3_ group is larger. However, this binding as well as the associated symmetrical bond is only weakly pronounced. The sp^2^CH_2_ (s) and sp^3^CH_2_ (as) oscillations also shift, but at higher wave numbers to 2971 cm^–1^ and 2932 cm^–1^, respectively.

After reaching the 120 nm a-C:H layer, the sp^2^CH_2_ (s) and sp^3^CH_2_ (as) oscillations shift back to their original values. In addition, the sp^3^CH_2_ (s) bond reappears weakly, but shifted (2858 cm^–1^). The (s) and (as) sp^3^CH_3_ oscillation are more pronounced here and the sp^3^CH_2_ (as) oscillation becomes the dominant one in the spectrum. The strengthening of the sp^3^ bond at this layer thickness is also reflected in the corresponding CAs, which decrease significantly compared to the 50 nm layer thickness. For the highest layer thickness of 170 nm, it is noticeable that the (as) oscillation of the sp^2^CH_2_ bond appears relatively strong at 3035 cm^–1^ [51]. Furthermore, there are only two small shift-backs (sp^3^CH_2_ (as) and (s)). The existing oscillations belonging to the sp^3^ bond are all comparatively more pronounced, but the proportion of sp^2^ bonds increases in line with CA values.

**Figure 7:**
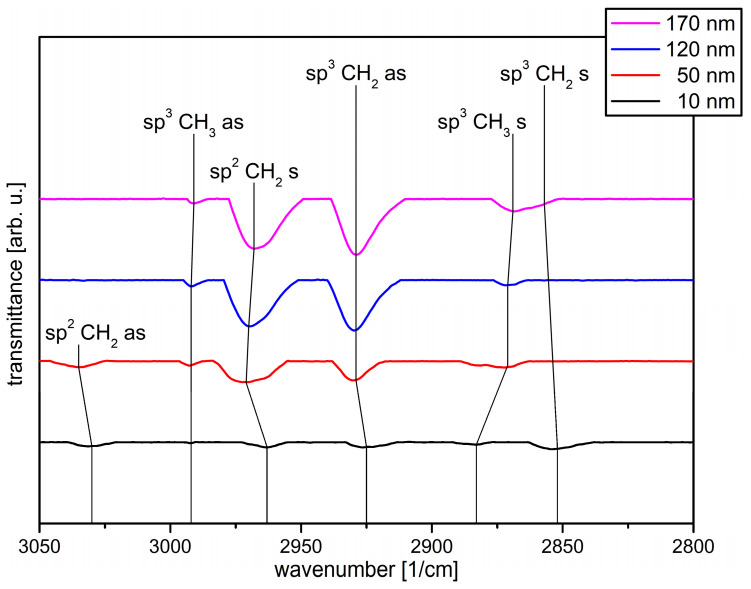
Analyzed DRIFT spectra of the “free” PHB samples coated with a-C:H from 10–170 nm in ascending order. The wavenumber is plotted against the transmittance in arbitrary units.

The DRIFT measurements were also performed for the “free” PHB samples and plotted in Figure 7. For the 10 nm a-C:H layer thickness it is noticeable that the sp^2^CH_2_ (as) binding is already present (3030 cm^–1^) in contrast to the “fixed” samples. The other five bonds, which could be seen for the “fixed” samples, are also present here, but slightly shifted. They are also only slightly accentuated due to the low layer thickness. However, the (as) and (s) bonds belonging to the sp^2^CH_2_ bond are not dominant here as in the “fixed” samples, but the oscillations are relatively equivalent (with the exception of the sp^3^CH_3_ (s) and (as) oscillations, which are only weakly visible).

If the layer thickness is increased to 50 nm, the symmetrical oscillation, which belongs to the sp^3^CH_2_ bond, disappears, similar to that in the “fixed” samples. In addition, the sp^2^CH_2_ (as) (from 3030 to 3035 cm^–1^), the sp^2^CH_2_ (s) (from 2963 to 2971 cm^–1^) and the sp^3^CH_2_ (as) oscillation (from 2925 to 2929 cm^–1^) shift to higher wave numbers. The sp^3^CH_3_ (as) bond remains unchanged in its position, the sp^3^CH_3_ (s) bond changes from 2883 to 2871 cm^–1^, whereby the peak of this oscillation is widened here. In total, the oscillations belonging to the sp^3^ bond dominate the spectrum, in accordance to CA values (Figure 5). With further increase of the layer thickness to 120 nm, the sp^2^CH_2_ (as) oscillation disappears. The associated symmetrical oscillation shifts slightly to 2970 cm^–1^. The sp^2^CH_3_ (s) oscillation is now sharper again, the dominance of the sp^3^ bond that was previously visible at 50 nm continues, and the CA as presented in Figure 5 decreases further.

If the a-C:H layer thickness exceeds 170 nm, the spectrum changes again compared to the 120 nm layer thickness. The sp^2^CH_2_ (s) bond changes from 2970 to 2968 cm^–1^ and the sp^3^CH_3_ (s) bond from 2971 to 2969 cm^–1^. In addition, the symmetrical oscillation belonging to the sp^3^CH_2_ bond reappears at a slightly different position of 2857 cm^–1^. The sp^3^ bonds are once again more strongly emphasized, which is also noticeable in the decreasing CA (Figure 5).

Overall, the chemical composition of the surface changes during the coating process for both “fixed” and “free” samples. The shift of the vibrations to higher/lower wave numbers is already known from other polymers and from previous measurements [11,13,16,48,49]. It is noticeable that the “free” samples at 10 nm have both the (s) and the (as) sp^2^CH_2_ oscillation, which is not the case for the “fixed” samples. Common to both systems is that with increasing film thickness, the sp^3^ components are initially more pronounced than the sp^2^ bonds. However, in the “free” samples one of the two sp^2^ oscillations (as) disappears after a layer thickness of 50 nm and the dominance of the sp^3^ bonds increases in the further course, whereas in the “fixed” samples the sp^2^ bond appears in higher layers and the proportion of the sp^2^ bonds increases. While the CA value for the “free” samples decreases from 120 to 170 nm, the CA for the “fixed” samples increases slightly, probably due to an increased sp^2^ content, which also appears in the DRIFT measurements. The measurements therefore complement and confirm each other.

## 4. Conclusions

The biopolymer PHB was coated with a-C:H layers of different thickness (10–170 nm) using C_2_H_2_ in an RF-PECVD plasma process. The polymer films were attached in two different ways: completely clamped on all four sides (“fixed”) and freely suspended with only one clamped side (“free”). The coated “free“ samples were examined with a profilometer for their bending radii and compared with the radii before. This allowed the stress present in the layers to be determined using Stoney’s equation. The resulting coatings were examined by SEM for surface morphology, CA for wettability and DRIFT for chemical composition of the surface.

Comparing SEM results, stress release phenomena, cord-buckling and layer failure occur in the “free” samples. This was due to a higher proportion of rigid sp^3^ bonds, as supported by CA and DRIFT. The results of the Stoney equation were also consistent with the SEM results. Although the layer thickness increased, the stress in the layer decreased, which was due to the increasingly pronounced stress release phenomena. The SEM images of the “fixed” samples show a homogeneous and closed layer. The two systems, “fixed” and “free”, differed chemically, as proven by DRIFT and CA. While the a-C:H in the “free” samples was more dominated by sp^3^ with increasing layer thickness and CA was consequently decreasing, the structure of the “fixed” samples seems to have changed in such a way that the sp^2^ bond was comparatively strengthened for higher layer thicknesses. Although the sp^3^ bond still dominated in CA, which was also decreasing, the sp^3^ bond decreased less strongly and the trend reversed at 170 nm with an even slightly increased angle. This higher sp^2^ content can be a reason for the stable and closed layers of the “fixed” samples detected in SEM, since a lower sp^3^ content leads to reduced stress.

Thus it is shown that the fixation of the samples during the coating process leads to different layers, although the “fixed” and “free” samples were coated simultaneously in the same plasma. While the “fixed” samples produced more stable films (no layer failure during the first 170 nm film thickness), the “free” samples underwent multiple stress release phenomena. A reorganization of the surface due to the method of sample attachment was therefore clearly visible.

## Data Availability

Data is contained within the article or Supplementary Material.

## Supplementary Materials

The following are available online at https://www.mdpi.com/2073-4360/13/2/184/s1; IR evaluation curve fitting by IR Solution–FTIR Control Software: Figures S1–S4; SEM images: reference, “fixed” and “free” samples.
