# Supplementary material for: Comparing the Influence of Residual Stress on Composite Materials Made of Polyhydroxybutyrate (PHB) and Amorphous Hydrogenated Carbon (a-C:H) Layers: Differences Caused by Single Side and Full Substrate Film Attachment during Plasma Coating
Source: Polymers (Basel). 2021 Jan 6;13(2):184. doi: 10.3390/polym13020184 (PMC7825619; doi:10.3390/polym13020184)
Supplement: Supplementary file 1 [file polymers-13-00184-s001.zip › SI-IR-PHB proofreading DONE.docx]

Supporting Information

Comparing the Influence of Residual Stress on Composite Materials Made of Polyhydroxybutyrate (PHB) and Amorphous Hydrogenated Carbon (a-C:H) Layers: Differences Caused by Single Side and Full Substrate Film Attachment during Plasma Coating

Torben Schlebrowski^1,^*, Rachida Ouali^2^, Barbara Hahn^2^, Stefan Wehner^1^ and Christian B. Fischer^1,3,^*

^1^ Department of Physics, University Koblenz-Landau, 56070 Koblenz, Germany; wehner@uni-koblenz.de (S.W.)

^2^ Department of Material Analysis, University of Applied Sciences Koblenz, RheinAhrCampus, 53424 Remagen, Germany; r_ouali@hotmail.de (R.O.); hahn@hs-koblenz.de (B.H.)

^3^ Materials Science, Energy and Nano-engineering Department, Mohammed VI Polytechnic University, 43150 Ben Guerir, Morocco

***** Correspondence: schlebrowski@uni-koblenz.de (T.S.); chrbfischer@uni-koblenz.de (C.B.F.); Tel.: +49-261-287-2365 (T.S.); +49-261-287-2345 (C.B.F.)

**List of contents.**

| **Page 1–3** | **IR evaluation curve fitting by**  **IR Solution–FTIR Control Software** |
| --- | --- |

IR evaluation curve fitting by IR Solution–FTIR Control Software

(Software version 1.30, Shimadzu Corporation, Kyoto, Japan)

| 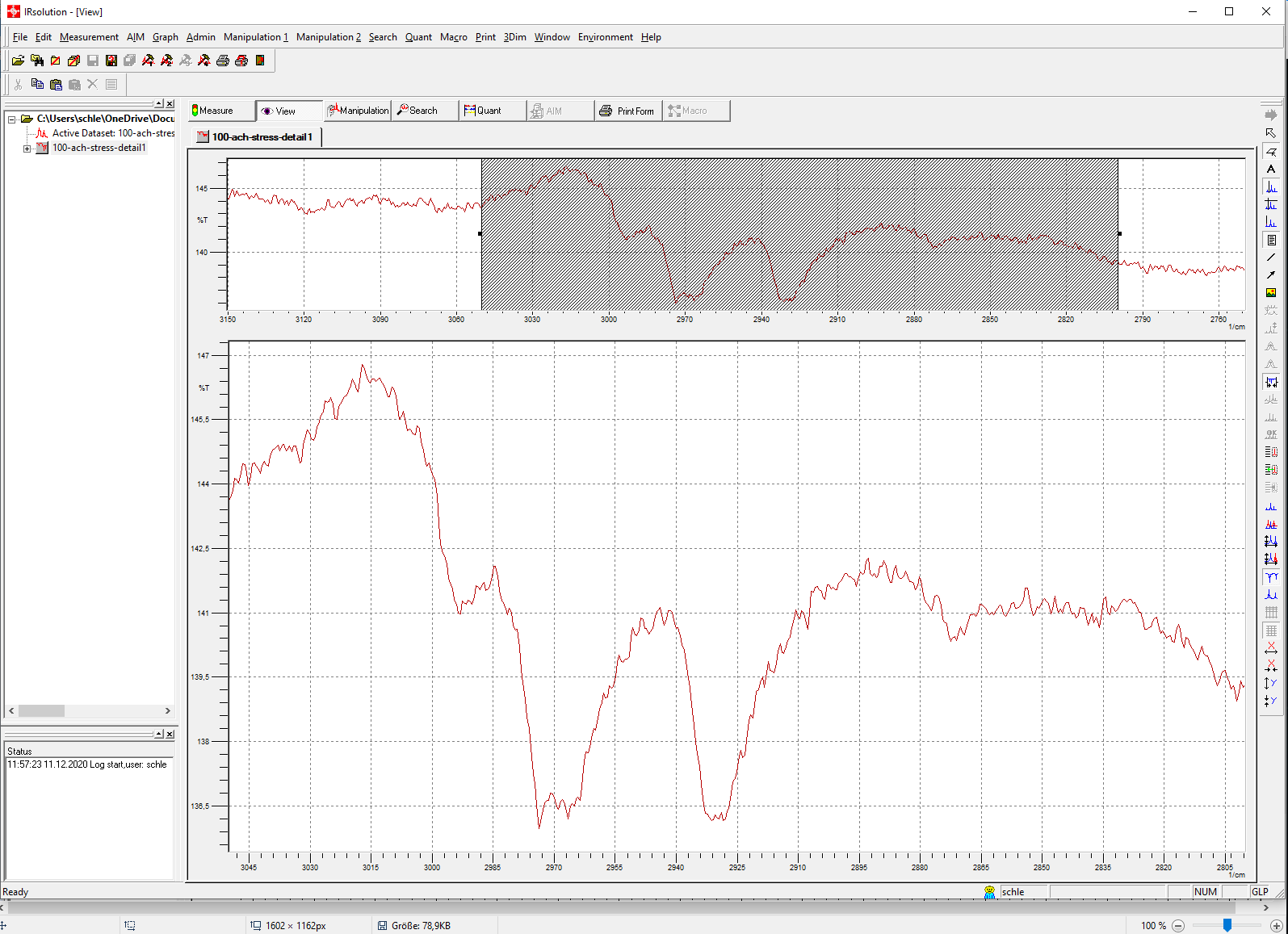 |
| --- |
|  |

**Figure 1.** Step 1: First of all, the peaks have to be determined by comparing all spectra recorded for the chosen sample (including all sample positions in detail + the full overview measurements).

|  |  |
| --- | --- |
| \| 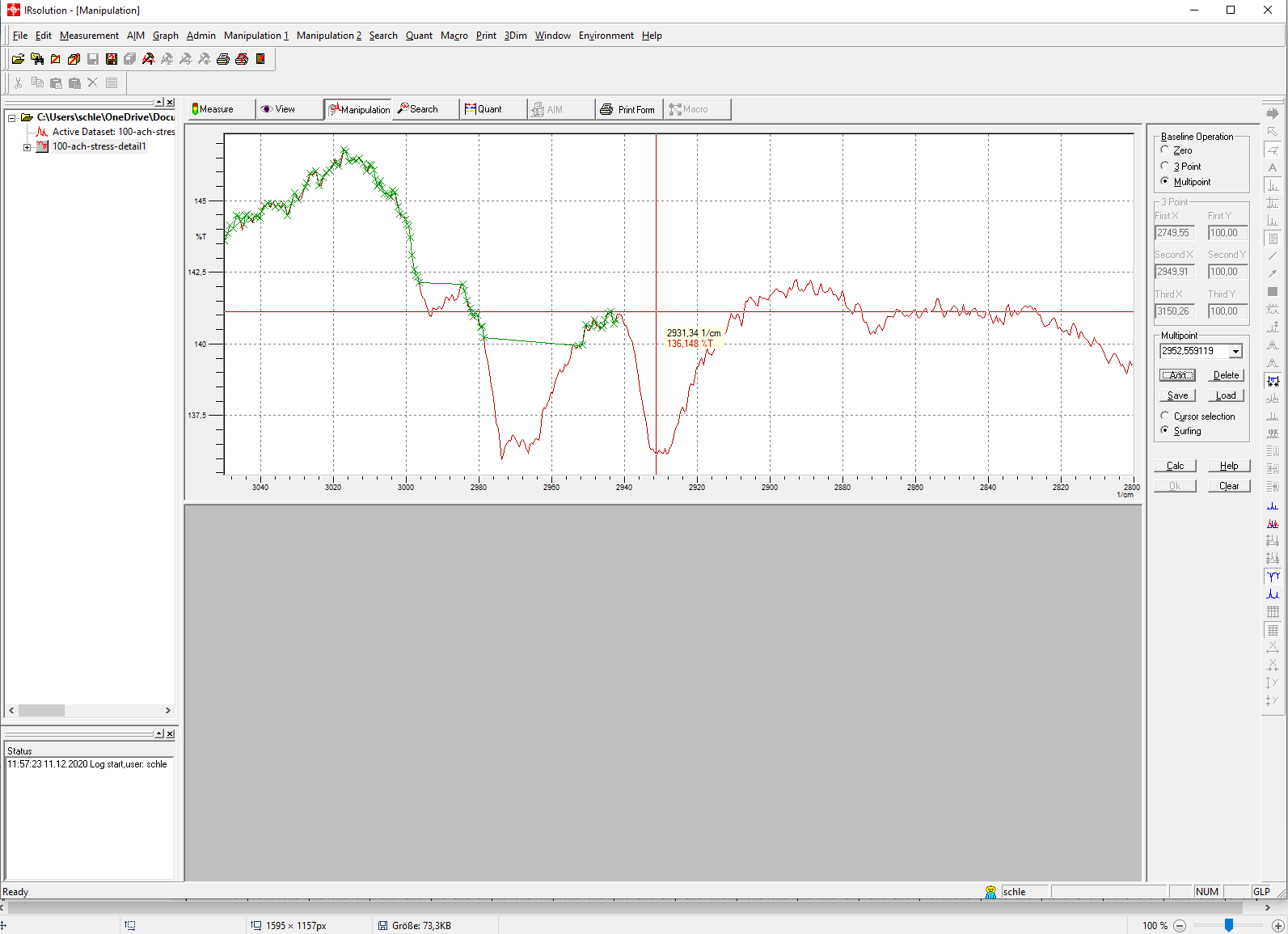. \| \| --- \| \|  \|   **Figure 2.** Step 2: Second step is the insertion of a multipoint baseline. Depending on how the individual points are placed for the multi-point baseline fit, the peak edges are somewhat more angular or rounder, but this does not change the statement of the peak position.   \| 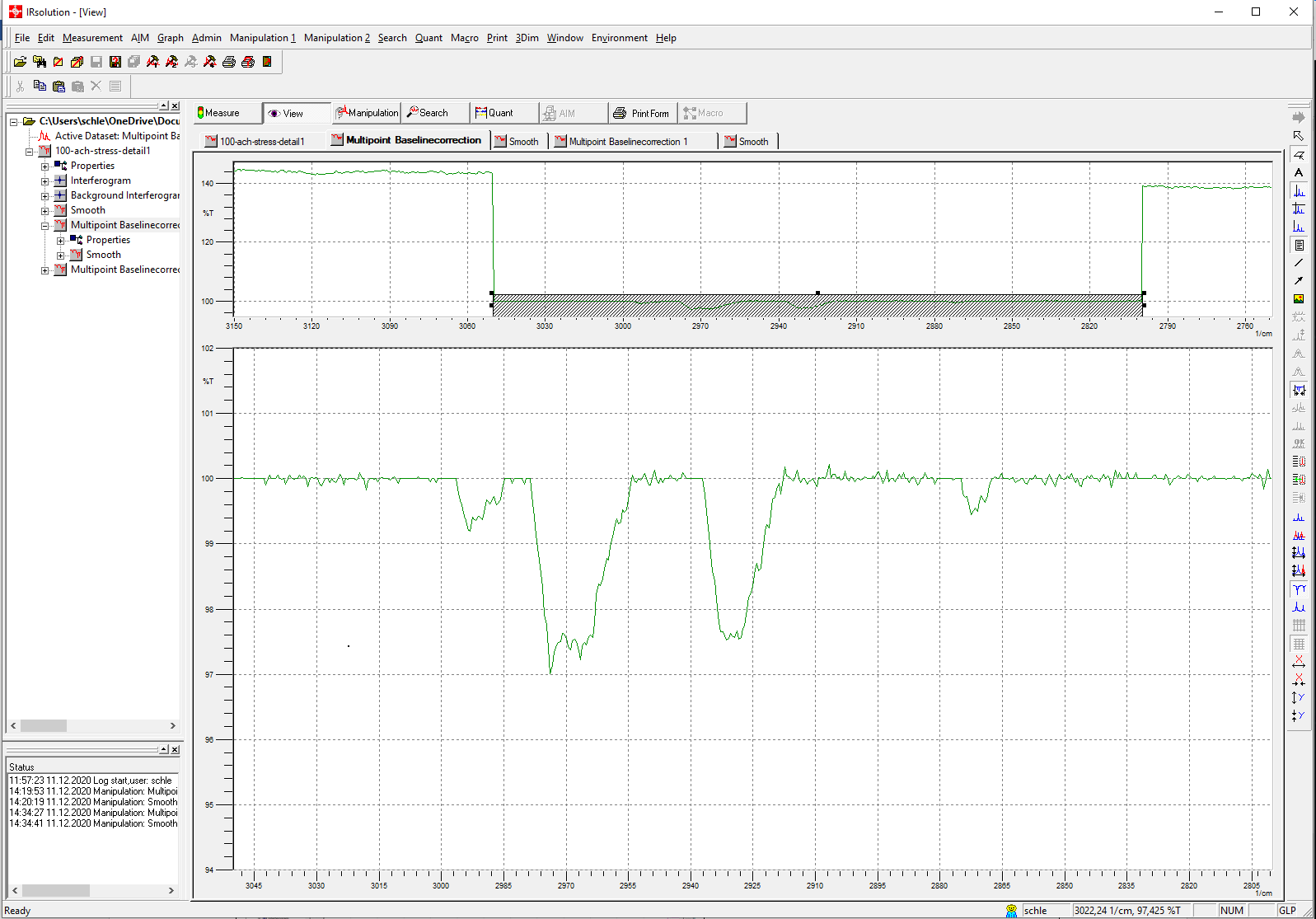 \| \| --- \| \|  \| |  |
| **Figure 3.** Step 3: After the multi-point baseline fit, the peaks that were measured are on a common baseline, which is still a little uneven. Therefore, a smoothing is performed in the following step.   \| 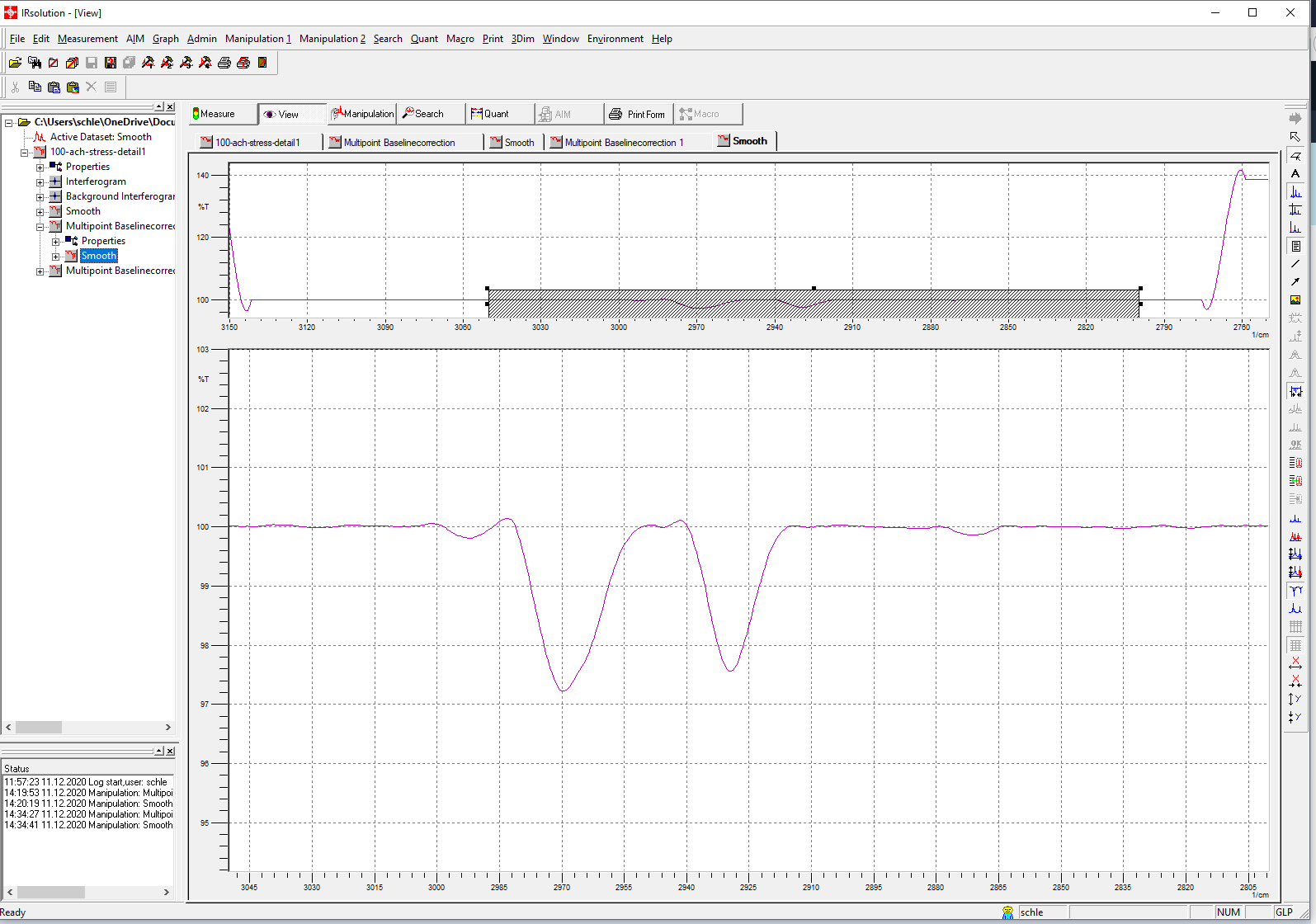 \| \| --- \| \|  \| |  |
| **Figure 4.** Step 4: As a result, a relatively smooth baseline is obtained with all measured peaks. Their position was not changed, the information content is constant. Only the peak starting points at the baseline can be slightly deformed by the previous multi-point baseline fit with many points manually set, without loss of peak information. |  |
|  |  |
