# Supplementary material for: Comparing the Influence of Residual Stress on Composite Materials Made of Polyhydroxybutyrate (PHB) and Amorphous Hydrogenated Carbon (a-C:H) Layers: Differences Caused by Single Side and Full Substrate Film Attachment during Plasma Coating
Source: Polymers (Basel). 2021 Jan 6;13(2):184. doi: 10.3390/polym13020184 (PMC7825619; doi:10.3390/polym13020184)
Supplement: Supplementary file 1 [file polymers-13-00184-s001.zip › SI-SEM-PHB proofreading-DONE.docx]

Supporting Information

Comparing the Influence of Residual Stress on Composite Materials Made of Polyhydroxybutyrate (PHB) and Amorphous Hydrogenated Carbon (a-C:H) Layers: Differences Caused by Single Side and Full Substrate Film Attachment during Plasma Coating

Torben Schlebrowski^1,^*, Rachida Ouali^2^, Barbara Hahn^2^, Stefan Wehner^1^ and Christian B. Fischer^1,3,^*

^1^ Department of Physics, University Koblenz-Landau, 56070 Koblenz, Germany; wehner@uni-koblenz.de

^2^ Department of Material Analysis, University of Applied Sciences Koblenz, 53424 Remagen, Germany; r_ouali@hotmail.de (R.O.); hahn@hs-koblenz.de (B.H.)

^3^ Materials Science, Energy and Nano-engineering Department, Mohammed VI Polytechnic University, 43150 Ben Guerir, Morocco

***** Correspondence: schlebrowski@uni-koblenz.de (T.S.); chrbfischer@uni-koblenz.de (C.B.F.); Tel.: +49-261-287-2365 (T.S.); +49-261-287-2345 (C.B.F.)

List of Contents

| **Page 1** | **SEM image: reference** |
| --- | --- |
| **Page 2–4** | **SEM images: fixed O_2_, 10, 50, 120 and 170 nm** |
| **Page 4–6** | **SEM images: free O_2_, 10, 50, 120 and 170 nm** |

Note: The deviations of the deposition heights in the designation of the SEM images come from the originally planned deposition height to the actually measured height (with profilometer) as resulting deposition.

SEM image reference

| 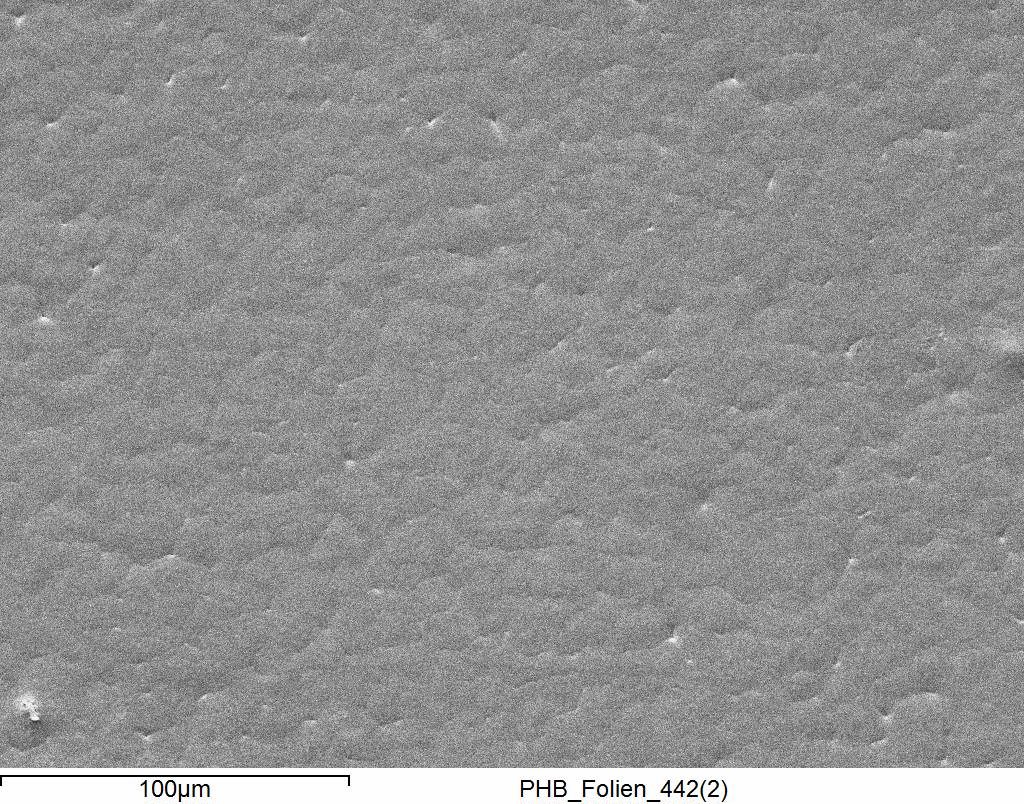 |
| --- |
|  |

SEM images “fixed”

| 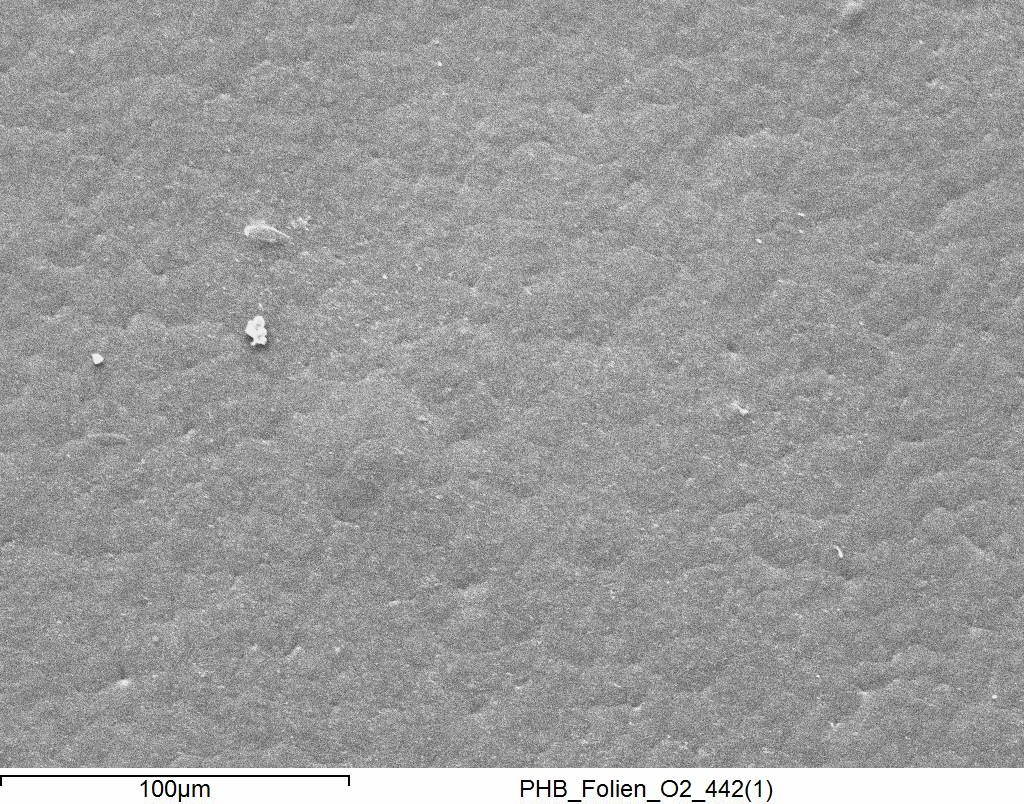 |
| --- |
|  |

| **O_2_ plasma treated**   \| 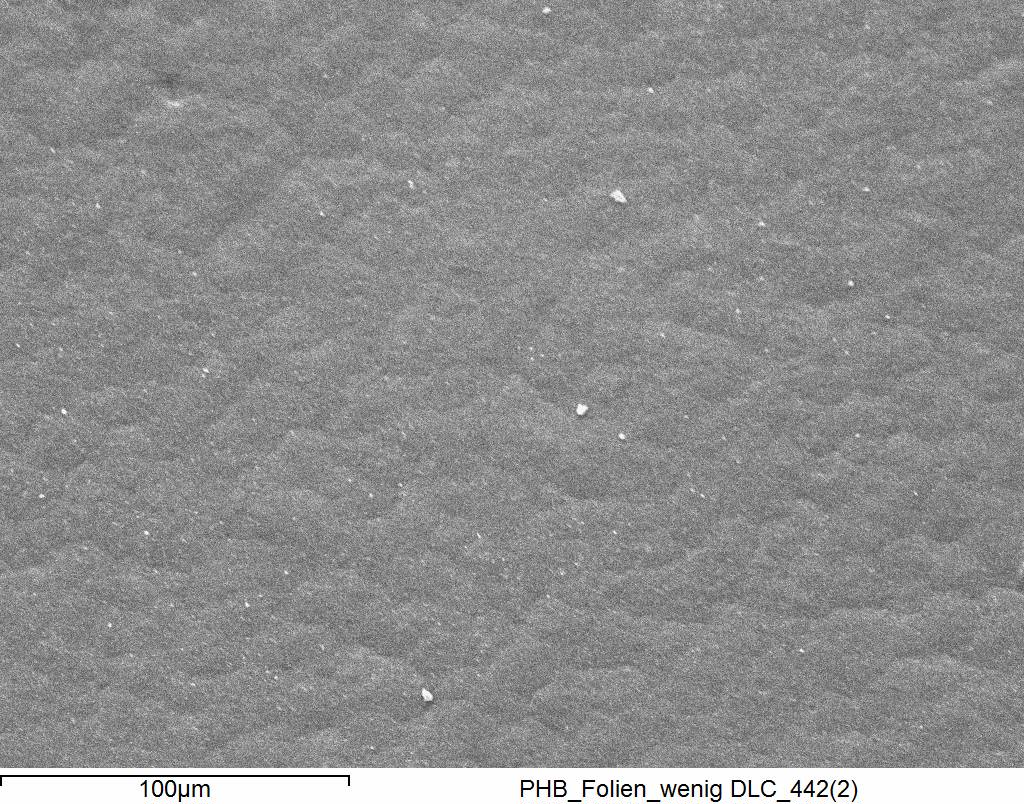 \| \| --- \| \|  \| | |  |
| --- | --- | --- | --- | --- |
| **10 nm a-C:H**   \| 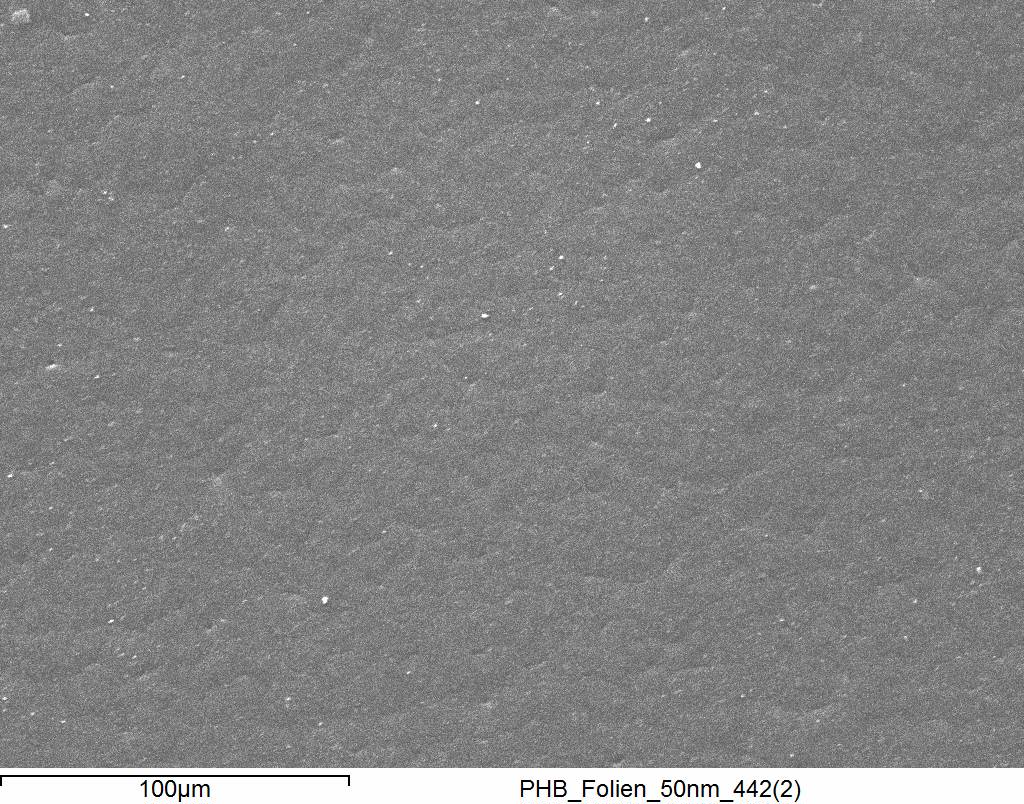 \| \| --- \| \|  \| | |  |
| **50 nm a-C:H**   \| 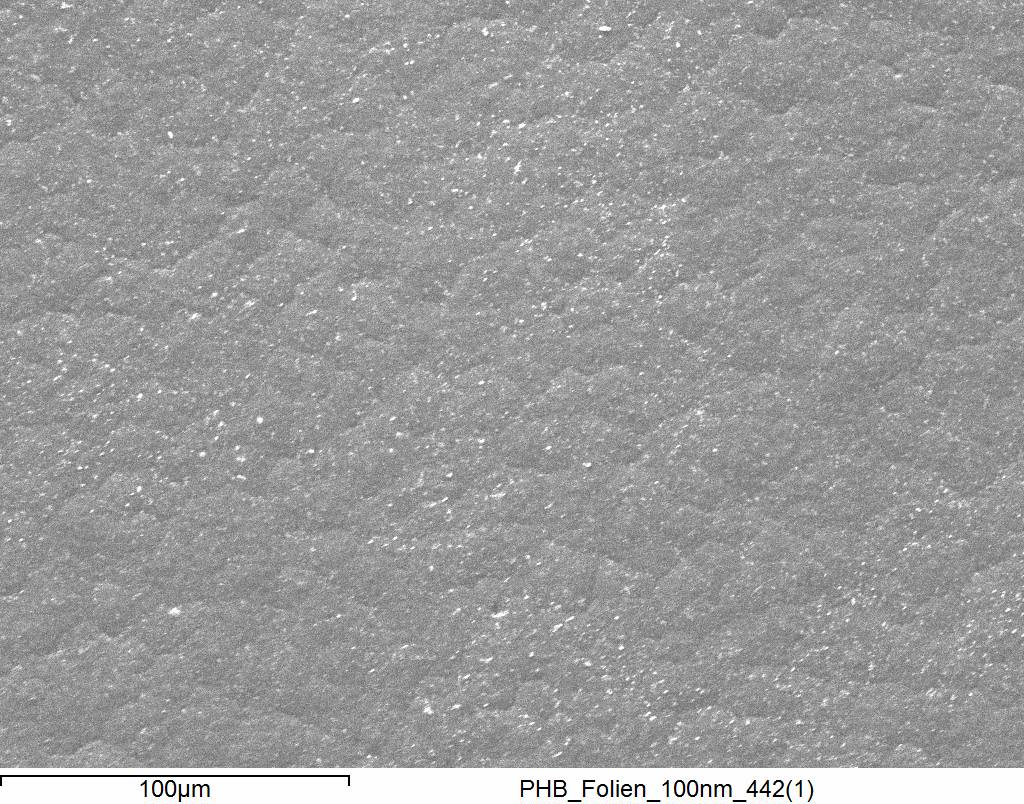 \| \| --- \| \|  \| | |  |
| **120 nm a-C:H**   \| 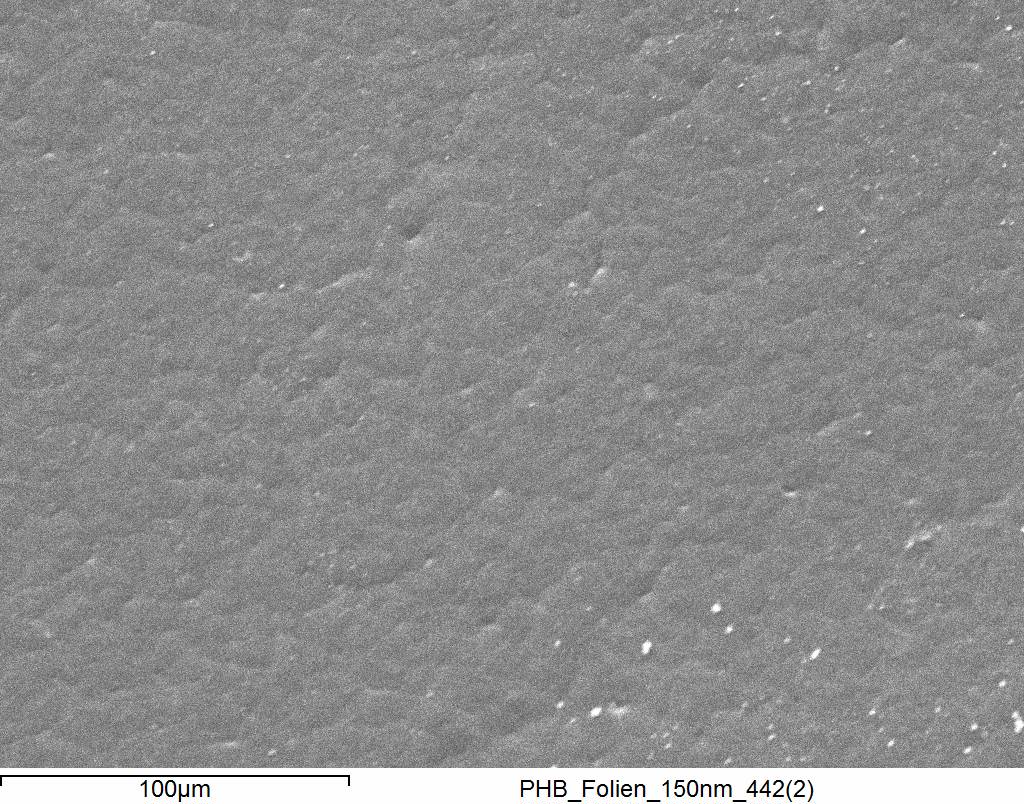 \| \| --- \| \| **170 nm a-C:H** \| | |  |
|  |  | |

SEM images “free”

| 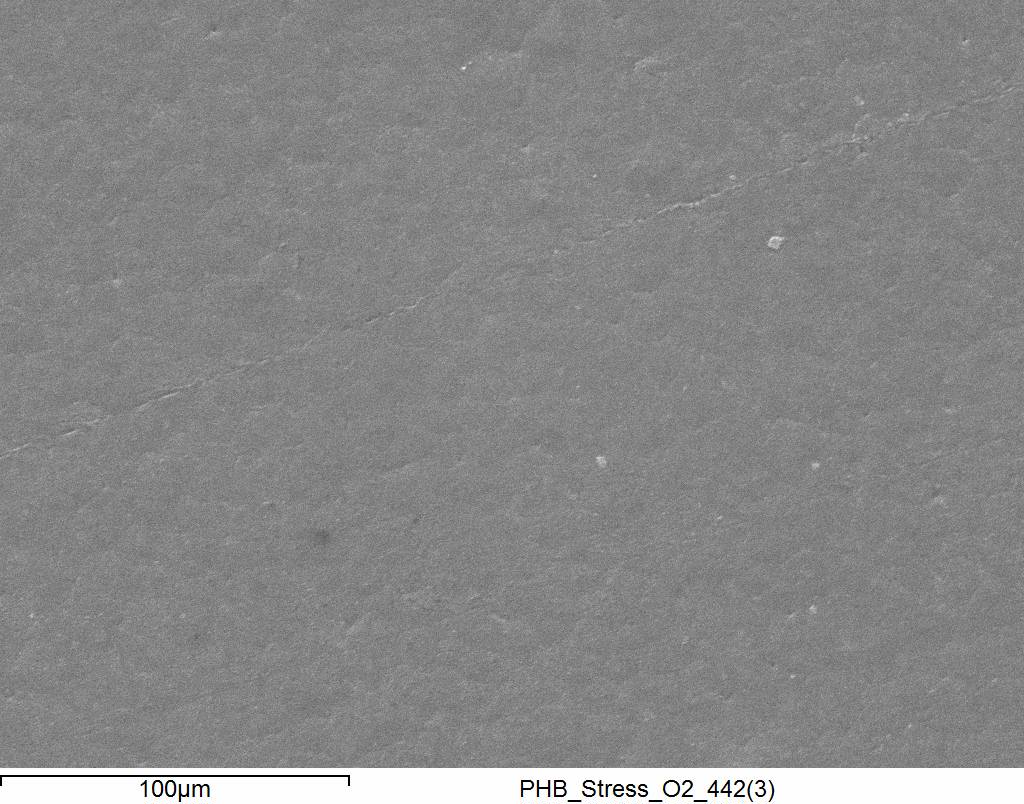 |
| --- |
|  |

**O_2_ plasma treated.**

| 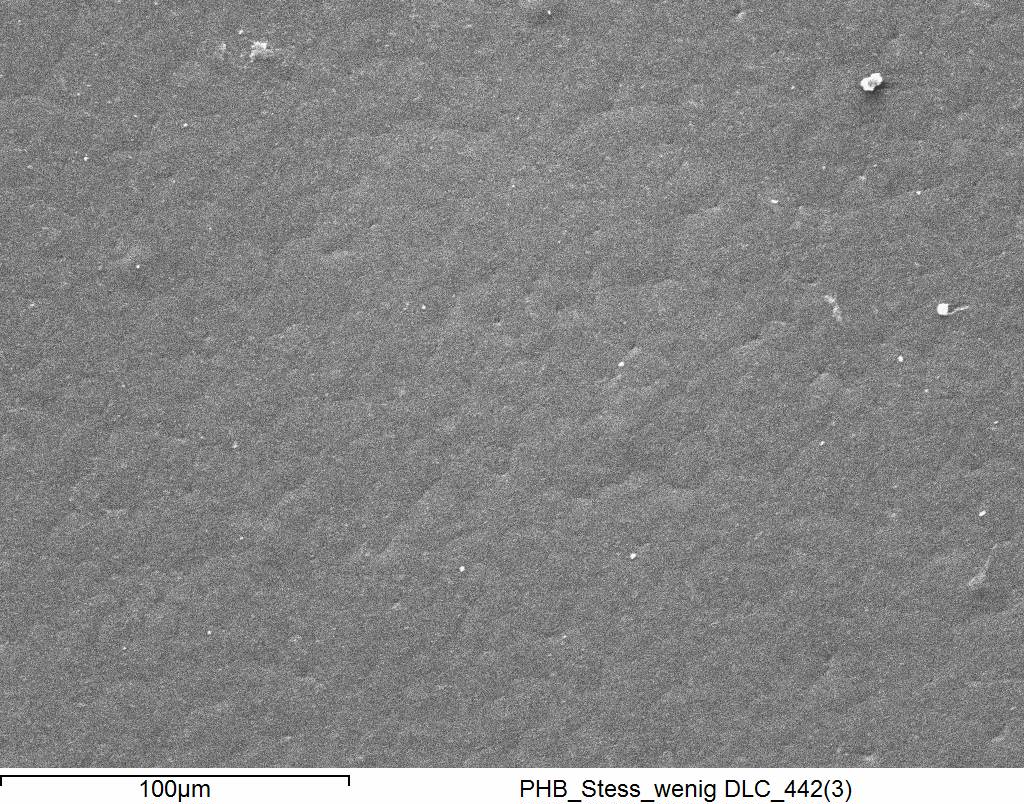 |
| --- |
|  |

**10 nm a-C:H**

| 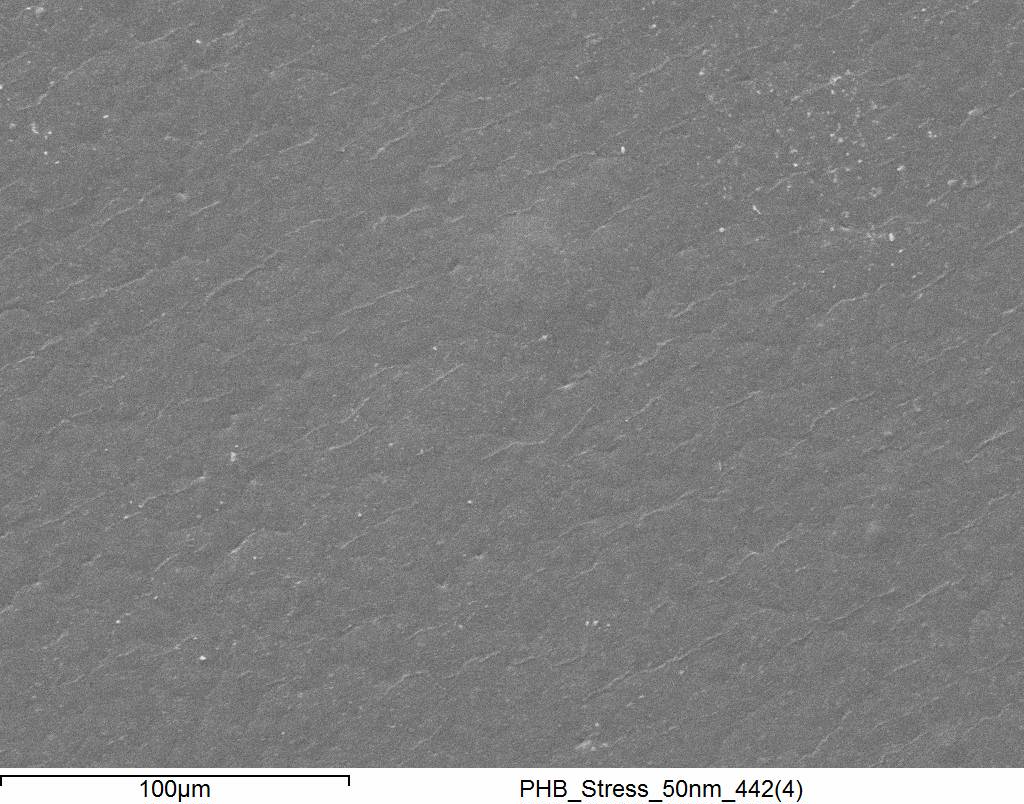 |
| --- |
|  |

**50 nm a-C:H**

|  |  |
| --- | --- |
| \| 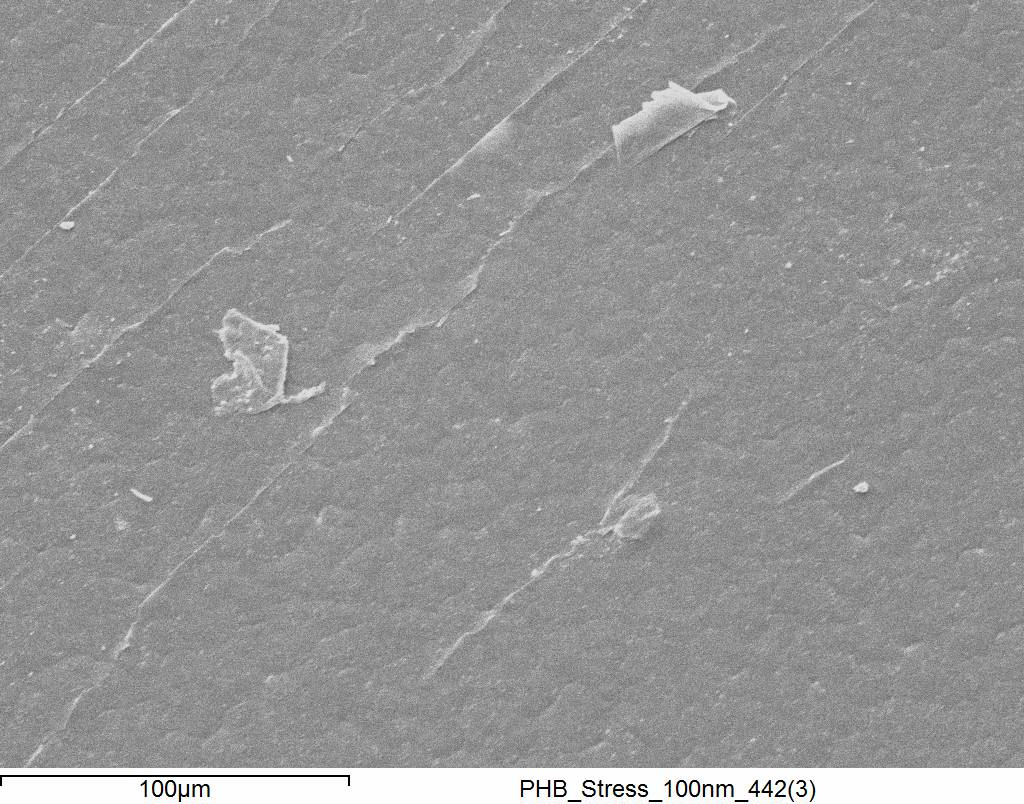 \| \| --- \| \|  \|   **120 nm a-C:H**   \| **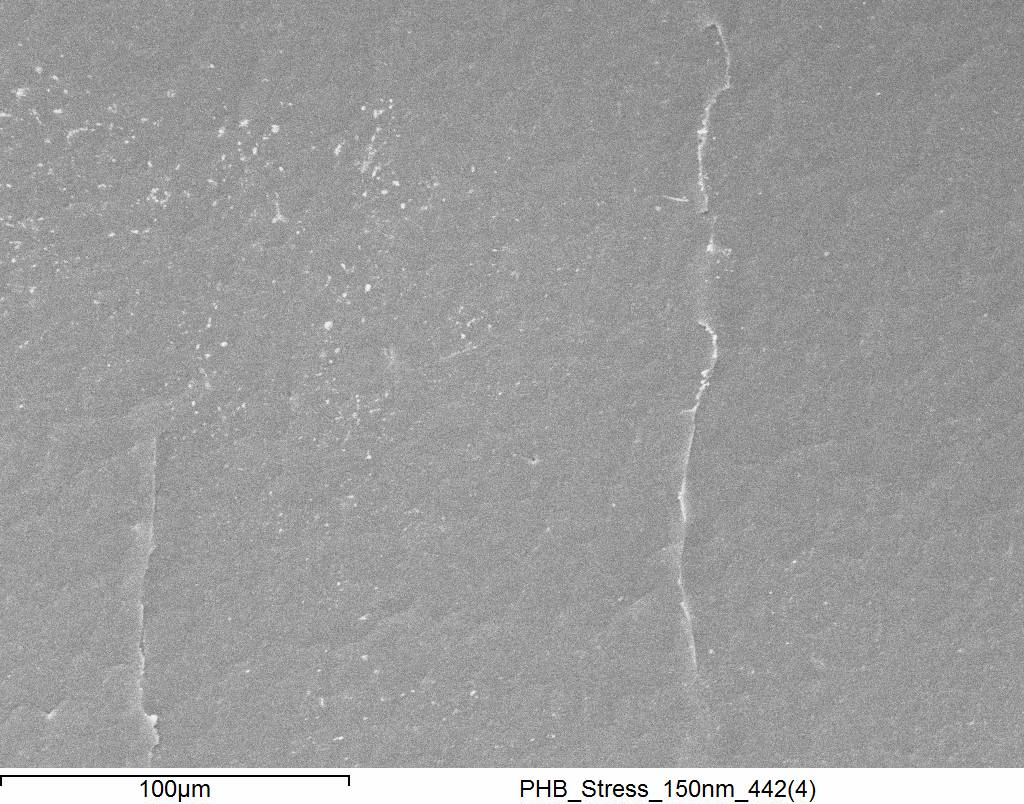** \| \| --- \| \|  \| |  |

**170 nm a-C:H**
